# Association between history of childbirth and chronic, functionally significant back pain in later life

**DOI:** 10.1186/s12905-022-02023-2

**Published:** 2023-01-03

**Authors:** Michelle Zhang, Corinne Cooley, Maisa S. Ziadni, Ian Mackey, Pamela Flood

**Affiliations:** 1 School of Medicine, Stanford University, Palo Alto, CA USA; 2 Present address: UW Internal Medicine Residency Program, 1959 NE Pacific Street, Seattle, WA 98195-6421 USA; 3 Stanford Health Care, Palo Alto, California USA; 4 Department of Anesthesiology, Perioperative and Pain Medicine, Stanford University, 300 Pasteur Dr, Palo Alto, CA 94305 USA

**Keywords:** Back pain, Pregnancy, Childbirth, Chronic pain, Acute pain

## Abstract

**Background:**

Back pain is more prevalent among women than men. The association with sex could be related to pregnancy and childbirth, unique female conditions. This association has not been thoroughly evaluated.

**Methods:**

Using a retrospective cohort design, we evaluated the relationship between history of childbirth on the prevalence and severity of functionally consequential back pain in 1069 women from a tertiary care pain management clinic. Interactions among preexisting, acute peripartum, and subsequent back pain were evaluated as secondary outcomes among the parous women using logistic and linear regression as appropriate.

**Results:**

The women who had given birth had a higher risk for functionally significant back pain compared to women who had not given birth (85% vs 77%, *p* < 0.001, Risk Ratio 1.11 [1.04-1.17]). The association was preserved after correction for age, weight, and race. Back pain was also more slightly severe (Numerical Rating Score for Pain 7[5-8] vs 6[5-7] out of 10, *p* = 0.002). Women who recalled severe, acute postpartum back pain had a higher prevalence of current debilitating back pain (89% vs 75%, Risk Ratio 1.19 (1.08-1.31), *p* = 0.001). Twenty-eight percent of acute postpartum back pain never resolved and 40% reported incomplete resolution.

**Conclusions:**

A history of pregnancy and childbirth is a risk factor for chronic functionally significant back pain in women. Severe acute postpartum back pain is a risk factor for future disability suggesting that the peripartum period may provide an important opportunity for intervention. Early recognition and management may mitigate future disability.

**Trial registration:**

The study was registered with clinicaltrials.gov as “Association Between Chronic Headache and Back Pain with Childbirth” (NCT04091321) on 16/09/2019 before it was initiated.

**Supplementary Information:**

The online version contains supplementary material available at 10.1186/s12905-022-02023-2.

## Introduction

Back pain is a leading cause of pain and disability with a similar incidence and prevalence worldwide [1–6]. According to the United States Center for Disease Control and Prevention, the lower back is the most common site of pain and a significant subgroup is treated with opioids [7–9]. The estimated economic burden of low back pain in the United States exceeds 84 billion dollars, depending on the research methodology [10–13]. The National Institutes of Health task force for chronic low back pain recommends that clinical studies characterize the “impact” of back pain by severity, pain interference with normal activities, and functional status [1, 14]. The task force anticipates that more precise characterization of back pain phenotype may lead to better understanding of specific risk factors for the development and worsening of these pathologies, leading to more effective strategies for prevention and intervention [15]. Strong correlations between physical, environmental, and psychosocial factors and chronic back pain associated with disability have been demonstrated in population-based studies [11, 16, 17]. Among these factors, female sex has been consistently associated with an approximately 13% elevated risk [18–20]. Women have obvious anatomical and physiological differences from men including the ability to bear children. The association between childbirth and chronic back pain has not been thoroughly evaluated.

Observational studies indicate that about 36-72% of pregnant women have acute back pain [21–23]. One study found that a significant proportion of women who report back pain during their pregnancies continue to experience recurring back pain with an associated reduction in their overall health [21]. Pregnancy is accompanied by weight gain, muscular redistribution, ligamentous relaxation, and change in the center of gravity [24–26]. These factors may contribute to the increased risk of chronic back pain in women. Unfortunately, most studies of postpartum back pain are limited to a few months to, at most, 2-3 years after delivery [27, 28]. Furthermore, while the interaction between acute back pain immediately postpartum with future back pain has been reported, few studies have evaluated the correlation between the acute postpartum back pain and the prevalence, severity, and functional impact of back pain years and decades later [29, 30].

We hypothesize that the prevalence of functionally significant back pain is greater in women who have given birth compared to those who have not after correcting for confounding variables. Secondarily, we posit that a history of childbirth is associated with more severe back pain and severe acute back pain after delivery. If a significant portion of the excess in back pain in women compared to men can be attributed to a history of pregnancy and childbirth, then the peripartum period could be a propitious moment for intervention. Immediately following delivery many women have access to health care and are focused on their future wellbeing. To address this gap in knowledge and consider avenues for intervention, we tested the hypothesis that a history of pregnancy and childbirth is associated with an increased likelihood of functionally significant back pain years and decades later.

## Methods

### Study design, setting and respondents

This retrospective observational cohort study was designed to compare the prevalence of functionally significant back pain between women who had and who had not given birth. The study was approved by the Stanford University Committee for the Protection of Human Subjects, Stanford, California, USA; Protocol: 52732; approved 23/08/2019. All methods were performed in accordance with the relevant guidelines and regulations. The study was registered with clinicaltrials.gov as “Association Between Chronic Headache and Back Pain with Childbirth” (NCT04091321) on 16/09/2019 before it was initiated. Enrollment occurred between 9/12/2019 and 26/2/2020. Women with chronic pain who had agreed to be contacted for research purposes (*n* = 12,776) were sent the email with the informed consent document and link to the questionnaire. All participants were women, as the study was designed to evaluate the impact of childbirth. The email title did not indicate a relationship to back pain or childbirth. The nature of the study was described only after the email was opened and informed consent was available. As such bias related to the topic of the study would only be applicable to those who opened the email. The email was opened by 7339 of the women and 1299 opened the questionnaire. Among those who opened the questionnaire, 1108 (85%) responded and 1069 (82%) provided complete data. The email included informed consent with a waiver of documentation as approved by the Stanford IRB. The standard consent was distributed along with a link to the questionnaire in an email that was distributed by Electronic Data Capture (RedCap) [31]. Women who did not respond to the email invitation were offered 4 additional opportunities approximately 1 week apart. All subject data are maintained on the secure RedCap electronic database hosted at Stanford University. Only the data on back pain are analyzed and presented here. Data related to the association between childbirth headache was planned as a secondary study and is not addressed in this manuscript.

### Primary outcome

The primary study hypothesis was that a history of pregnancy and childbirth would be associated with a higher prevalence of functionally significant back pain after adjusting for patient characteristics that have previously been associated with back pain; age, weight, ethnicity, race, employment, and family income [4, 11, 32–35]. The primary outcome variable, prevalence of functionally significant back pain, was defined as a simple yes/no response to the question, “Have you ever had back pain that prevented you from doing the things that you need to do?” This question has been used to indicate back pain with functional significance to our patients in two of our prior studies [36, 37]. The exposure variable, prior pregnancy and childbirth, were defined as a binary “yes/no” response to the question, “Have you given birth yourself?”

### Secondary outcomes

There were three preplanned secondary outcomes.

1. Back pain severity
  Exposure: prior pregnancy as defined above
  Population at risk: All subjects reporting back pain (e.g., NRS > 0)
2. Prevalence of backpain
  Exposure: acute severe backpain within 5 days of delivery
  Population at risk: Women who had given birth
3. Back pain severity
  Exposure: acute severe backpain within 5 days of delivery
  Population at risk: Women who had given birth and reported back pain (NRS > 0) in later life.

The secondary outcomes 2 and 3 assessed for association between neuraxial anesthesia for labor and delivery, time to resolution of postpartum back pain, and patient characteristics as noted above.

### Sample size calculation

There was no a priori sample size calculation as the available population was fixed. As noted above, 1069 women provided complete data. Of these 204 women did not have back pain, and 865 women had back pain. The study therefore had 99% power to detect a difference in proportion of “medium effect size” (i.e., Cohen’s h = 0.5).

### Data analysis plan

The relationship between a history of pregnancy and childbirth with back pain was analyzed and adjusted for back pain including age, weight, employment, race, ethnicity, and family income using logistic regression. The primary outcome variable was the association of a history of pregnancy and childbirth with functionally significant back pain in the logistic model. Statistical significance for the primary outcome was set at *p* < 0.05.

The secondary outcome variables were only assessed to guide future research, and not to demonstrate significant associations. *P* values and relative risks for all secondary outcomes are provided only as measures of the strength of the association and are not intended to infer statistical significance.

The association of binary variables (e.g., prior pregnancy and childbirth) to the primary outcome is expressed as relative risk (RR) with 95% confidence interval, presented as RR [95% CI]. Relative risk was calculated using unconditional maximum likelihood estimation. Confidence intervals were calculated using normal approximation using the epitools package version 0.5-10.1 in R programing language.

### Model creation

The primary logistical model was estimated using forward selection to include only variables with significant associations with the primary outcome variable at alpha < 0.05. During model creation, variables that did not retain a *p*-value < 0.05 were excluded. The impact of childbirth on backpain severity was evaluated similarly with linear regression. We excluded missing values at the beginning of analyses so that the sample size remained constant. Statistical analyses were completed using R programming language version 4.0.3 (“Bunny-Wunnies Freak Out”, 2020-10-10). Additional R packages are discussed in the descriptions of the statistical tests. The figures were created with the ggplot2.

## Results

**Fig. 1:**
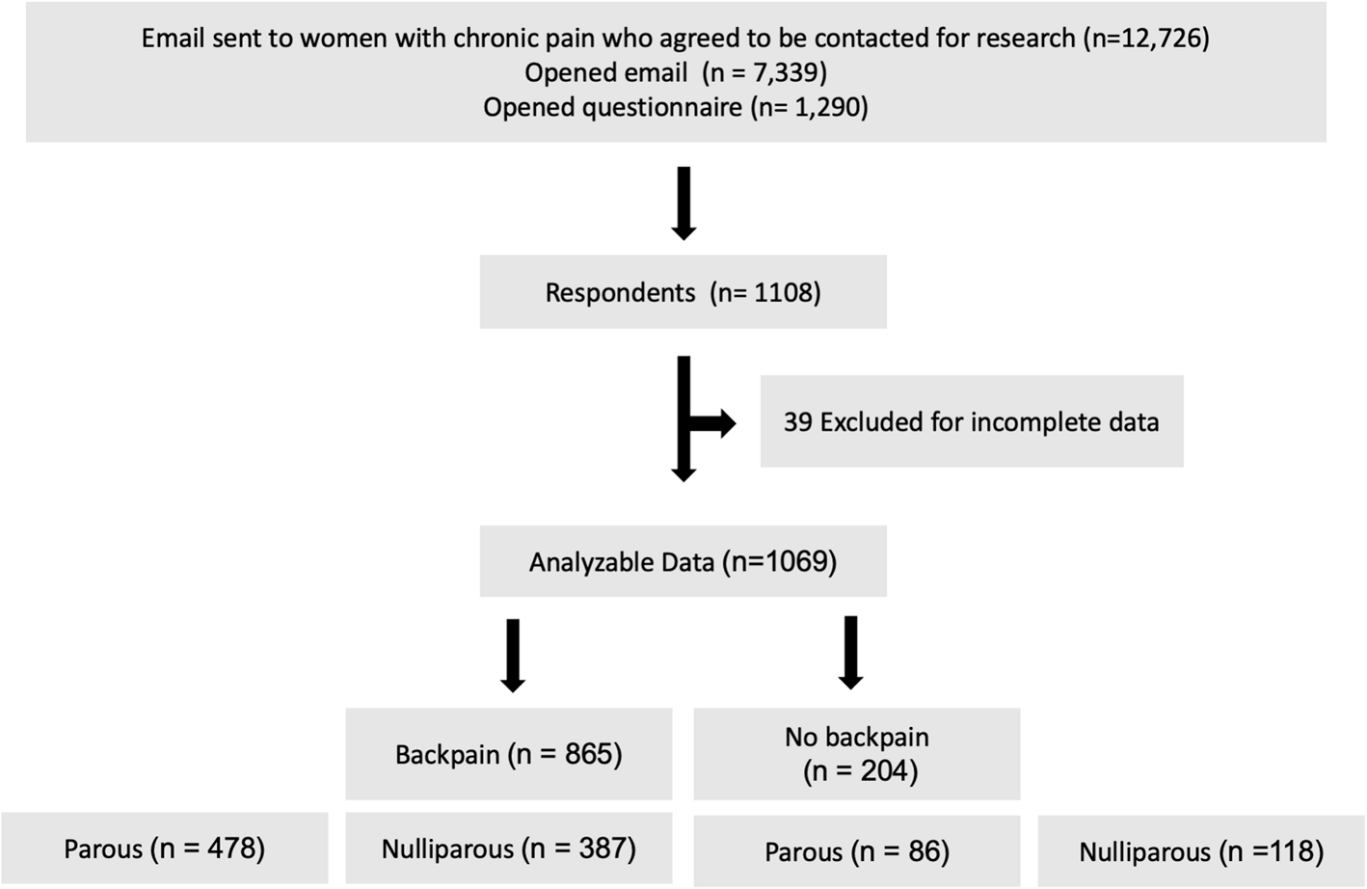
Flow diagram from email solicitation for participation to completed data set

Figure 1 shows the patient flow through the study. Participant characteristics are presented in Table 1. The women who had given birth had done so 29 [20-39] years previously.

**Table 1:** Participant characteristics

	(***N*** = 1069)
**Age (years)**	52 [38, 62]
**Weight (kilograms)**	73 [61, 86]
**Parous**	564 (53)%
**Race**	
African American	45 (4) %
Asian	95 (9) %
Caucasian	806 (75) %
Native Hawaiian or Pacific islander	11 (1) %
Native American	33 (3) %
Prefer not to state	18 (2) %
Other	127 (12) %
**Ethnicity**	
Hispanic	144 (14) %
**Income**	
< 20 k	99 (9) %
20 k-35 k	97 (9) %
35 k-150 k	452 (42) %
150 k-200 k	120 (11) %
> 200 k	134 (13) %
Prefer not to state	114 (12) %
Missing	53 (5) %

Median [IQR – Interquartile range]

Count (% - percent)

### Primary outcome variable: association between history of pregnancy and childbirth and lifetime prevalence of functionally significant back pain

Pregnancy and childbirth were associated with increased prevalence of functionally significant back pain compared that in women who have not given birth (85% vs 77%, *p* < 0.001, RR 1.11[1.04 -1.17]). Before adjustment, women who reported back pain were older (53 [40-63] vs 49 [33-59] years, *p* = 0.003) and heavier (73[63-88] vs 67[59-80] kg, *p* < 0.001). The prevalence of significant back pain in Asian women was 66%, which was lower than the 82% prevalence in non-Asian women (*p* < 0.001, RR 0.81 [0.70 - 0.93]). There was no difference in the relative risk of back pain associated with other patient characteristics including other races, ethnicity, and reported income. After adjustment for these covariates significant at the 0.05 level with multivariable logistic regression, pregnancy and childbirth (*p* = 0.001), greater weight (*p* = 0.001), and non-Asian race (*p* = 0.002) remained as positive predictors for debilitating back pain. Age was no longer associated with the prevalence of back pain (see supplement A for regression details).

### Secondary outcome variable: association between childbirth and current back pain severity

**Fig. 2:**
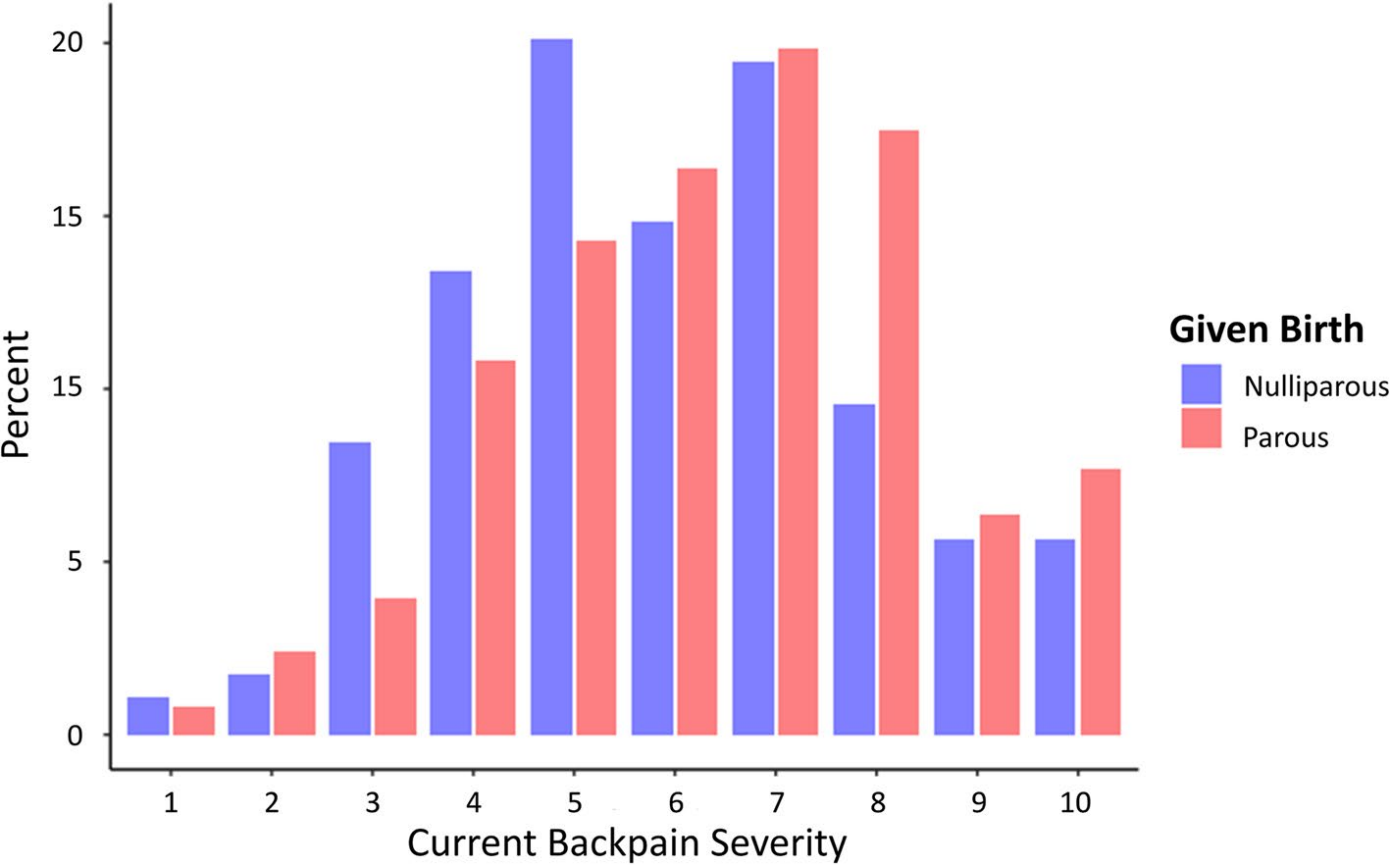
Relationship between parity and backpain severity in later life. Women who had given birth had more severe backpain (NRS 7[5-8] vs 6[5-7] out of 10, *p* = 0.002). NRS – Numerical rating scale for pain, [Interquartile Range]

Parous women reported slightly more severe back pain compared to women who had not given birth (NRS 7[5-8] vs 6[5-7] out of 10, *p* = 0.002, Fig. 2). Race (*p* < 0.001), ethnicity (*p* = 0.004), and family income were individually associated with back pain severity. The most significant racial difference was between White and non-White women (NRS 6[5-7] vs 7[5-8]). Hispanic women reported more severe back pain than non-Hispanic women (NRS 7[5-8] vs 6[5-7]. There were two major break points in the interaction between family income and pain severity. Women who reported a family income of less than $20,000/year reported back pain NRS 7[6-9]. Those who reported family income between 20 and 50,000/year reported back pain with NRS 6[5-8]. Those with family income of more than $50,000 reported back pain with NRS 6[6-7]. Age and weight were not associated with severity. After adjustment for the above significant covariates with multivariable linear regression, only having given birth (*p* = 0.002) and non-white race (*p* < 0.001) remained as predictors for back pain severity (see supplement B for regression details).

### Association of back pain prevalence and severity with postpartum back pain and other childbirth related factors

Thirty-three percent of women with an independent recollection reported severe acute postpartum back pain within a week of delivery. The postpartum back pain was recalled as 7[6-9] out of 10 in severity. Those who recalled severe acute postpartum back pain had a higher prevalence of current functionally significant back pain (89% vs 75%, RR 1.19[1.08-1.31], *p* = 0.001).

The women who reported severe acute postpartum back pain began having functionally significant back pain at a younger age (27[20,38] vs 40[30,54], *p* < 0.001), were more likely to have had back pain prior to delivery (46% vs 18%, *p* < 0.001; RR 2.58[1.80-3.69]), and were more likely to be Hispanic (21% vs 10%, *p* = 0.003; RR 2.16[1.31-3.54). There were no differences with respect to their age at first birth, weight, race, family income, or use of neuraxial anesthesia. Thirty-two percent of women with severe acute back pain gave birth by cesarean section.

### Resolution of acute postpartum back pain

Twenty-eight percent of the women with acute postpartum back pain reported that the back pain never resolved, 40% reported partial resolution, and 31% reported complete resolution. Among those who reported that their back pain had at least partially resolved, 59% resolved within 1 year, 27% between 1 and 5 years, and 14% reported resolution after more than 5 years.

## Discussion

Our findings support our primary hypothesis that a history of pregnancy and childbirth is positively associated with an increased prevalence and severity of functionally significant back pain among chronic pain patients. In our study, the median interval between delivery and contemporaneous report of back pain was 29 years. The association between functional limitation and pregnancy and childbirth remained significant after correcting for age and race that were different between groups. Our study contributes to the current literature by providing evidence that pregnancy is associated with a risk of functionally significant back pain, decades after women have given birth. Our findings are supported by both a previous study that demonstrated association between the number of previous pregnancies and persistent back pain up to 6 months after childbirth, and another cross- sectional study that found that 68% of women who had back pain during pregnancy reported low back pain later in life [21, 38].

Peripartum back pain is common but typically self-limited. Previously, there has been no consensus that it is related to future back pain outside the context of the puerperium [27, 39]. Herein we have documented that 14% of women with acute postpartum back pain did not have resolution more than 5 years later. Our results are consistent with the findings of Norén and colleagues who estimated that 20% had residual back pain 3 years later [27].

Given that women commonly receive medical care at this time, new or acutely worsened back pain immediately after delivery may be an important opportunity for clinical intervention. It may be a “teachable moment” when women interact intensely with the medical community and may be open to education and intervention for their future wellbeing. Identifying women at risk for long-term back pain and teaching them the importance of prevention and mitigation with non-pharmaceutical methods may be particularly impactful at this time in life.

Treatment may be targeted to high risk groups such as women with severe acute postpartum back pain, as our findings suggest that these women had a higher risk for severe functionally significant back pain in later life. Intervention during pregnancy or in the early postpartum period may reduce both acute peripartum back pain and prevent the development of chronic back pain and disability. Non-pharmacological approaches would be valuable for future study and could replace the common reflex to prescribe opioids.

Unfortunately, most patients who delivery by cesarean section and a minority of those who have a vaginal delivery are treated with opioids immediately postpartum in the United States [40]. This can be the onset of a treatment that is typically inappropriate for chronic musculoskeletal back pain. Future implementation studies of nonmedicinal treatments that have good evidence and are recommended by both obstetricians and physical therapists are in order.

Indeed, the American College of Obstetrics and Gynecology recommends that women with healthy, uncomplicated pregnancies engage in at least 20 to 30 minutes of moderate physical activity on most or all days of the week during their pregnancy and postpartum [41]. Recently published guidelines emphasize an important role for physical therapy in managing postpartum back pain [42, 43]. However, the guidelines have not yet been widely adopted.

Both psychological and physical therapy treatments are known to reduce pain and the need for opioids in patients with back pain [27, 30, 44–46]. They are useful in women who experience acute back pain following labor and delivery, and thus may prevent progression to chronic back pain and opioid use if implemented in the peripartum period. These are important avenues for future study.

Our study has important limitations that are part and parcel of all retrospective study designs. Back pain is a complex syndrome that usually has multiple causes. Childbirth is unlikely to be entirely responsible for back pain in any of the women in our study. Clearly women are different from men in many ways unrelated to pregnancy that may contribute to back pain including anatomical differences, with greater curvature of their spine, a caudally located lordotic peak, and significant changes related to hormonal withdrawal during menopause and many other factors [34, 47, 48]. However, the strength of association that we identified in our study is substantial and similar or larger in strength and size to well recognized associations between back pain and age, weight, race, and ethnicity [4, 11, 32–35, 49]. These known covariates function as an internal control on our methodology and validate the association we found. Another limitation is our use of a simple binary description of functionally significant back pain and disability: “… back pain that prevented you from doing the things that you need to do”. We acknowledge that back pain is a complex construct, and it is possible that patients in our survey who responded “yes” may not meet other criteria for chronic back pain. We have used this definition here and in our previous studies to identify salience to the individual [36, 37].

We had a relatively low response rate to our survey that might indicate response bias. However, while the contact email was sent to a large number (12,776) of women who indicated that they are willing to be contacted for research by our pain management clinic, the subject line did not indicate the nature of the study. Stanford Pain Medicine maintains a large data base of individuals who indicate their willingness to be contacted for any research project. The 5437 recipients who did not open the email could not have made their decision based on personal interest in the topic because the information was not available. The topic of the study was of course apparent on opening the email and reading the informed consent. A response bias could also be another type of chronic pain.

As noted above, all the women who responded to our survey had had contact with an academic chronic pain clinic and had agreed to be contacted for pain research. As such they may not be representative of the population at large. Our findings are potentially valuable to the many women with chronic pain conditions considering pregnancy and those currently at risk for progression from acute postpartum to chronic back pain who could benefit from nonpharmacological treatment. Future work in a low risk cohort could provide insight that is more generally applicable.

## Conclusions

Women are more likely than men to experience chronic back pain during their lifetime. Our data suggest that a history of childbirth may account for up to 10% of the excess risk in women compared to men. Future outcome studies on the impact of various non-pharmacological treatment methodologies including peripartum physiotherapy and pain psychology especially directed to women at risk would be valuable to identify procedures to reduce the excess of chronic back pain and related disability in women.

## Supplementary Information

**Additional file 1: Supplementary figure.** Regression Model Details.

## Data Availability

The datasets generated and analyzed during the current study are not publicly available as they contain data to be published in a future secondary study but are available from the corresponding author on reasonable request.

## Abbreviations

NRS: Numerical rating scale for pain
RR: Relative risk
IQR: Interquartile range
K: Thousand
%: Percent
