# Supplementary material for: Association between history of childbirth and chronic, functionally significant back pain in later life
Source: BMC Womens Health. 2023 Jan 3;23:4. doi: 10.1186/s12905-022-02023-2 (PMC9809019; doi:10.1186/s12905-022-02023-2)
Supplement: Supplementary file 1 — Additional file 1: Supplementary figure. Regression Model Details. [file 12905_2022_2023_MOESM1_ESM.pdf]

A. Primary outcome variable- Back Pain and History of Childbirth

	Model 1					Model 2					Model 3				
**Parameters**	**β**	**SE**	**p - value**	**95% CI**	**AIC**	**β**	**SE**	**p - value**	**95% CI**	**AIC**	**β**	**SE**	**p - value**	**95% CI**	**AIC**
**Intercept**	1.188	0.105	< 0.001	0.985, 1.398	1035	0.706	0.263	0.007	0.019, 1.226	1033	-0.518	0.412	0.209	-1.330,0.285	1019
**Childbirth**	0.528	0.157	< 0.001	0.220, 0.838		0.414	0.168	0.0136	0.086, 0.745		0.391	0.17	0.021	0.060, 0.725	
**Age**						0.011	0.006	0.0494	0.000, 0.022		0.011	0.006	0.052	0.000, 0.022	
**Weght**											0.017	0.004	<0.001	0.008, 0.026	
**Asian Race**															

	Model 4					Model 5				
**Parameters**	**β**	**SE**	**p - value**	**95% CI**	**AIC**	**β**	**SE**	**p - value**	**95% CI**	**AIC**
**Intercept**	-0.213	0.427	0.619	-1.055, 0.622	1014	0.193	0.345	0.576	-0.491, 0.863	1014
**Childbirth**	0.430	0.171	0.012	0.0955, 0.766		0.525	0.160	0.001	0.213, 0.840	
**Age**	0.009	0.006	0.111	-0.002, 0.0199						
**Weght**	0.015	0.005	0.001	0.006, 0.024		0.015	0.005	0.001	0.006, 0.024	
**Asian Race**	-0.682	0.242	0.005	-1.150, -0.198		-0.732	0.240	0.002	-1.196, -0.253	

A. Secondary outcome variable- Back Pain Severity and History of Childbirth

	Model 1					Model 2				
**Parameters**	**β**	**SE**	**p - value**	**95% CI**	**R^2**	**β**	**SE**	**p - value**	**95% CI**	**R^2**
**Intercept**	5.862	0.127	<0.001	5.612, 6.111	0.017	6.39	0.19	< 0.001	6.017, 6.761	0.037
**Childbirth**	0.558	0.168	<0.001	0.229, 0.887		0.525	0.166	0.002	0.199, 0.851	
**White Race**						-0.691	0.186	< 0.001	-1.057, -0.326	
**Hispanic Ethnicity**										
**Income**										

	Model 3					Model 4				
**Parameters**	**β**	**SE**	**p - value**	**95% CI**	**R^2**	**β**	**SE**	**p - value**	**95% CI**	**R^2**
**Intercept**	6.214	0.216	< 0.001	5.790, 6.638	0.040	6.283	0.226	< 0.001	5.839, 6.727	0.041
**Childbirth**	0.541	0.166	0.001	0.215, 0.867		0.536	0.166	0.001	0.210, 0.863	
**White Race**	-0.553	0.203	0.007	-0.952, -0.154		-0.539	0.204	0.008	-0.940, -0.139	
**Hispanic Ethnicity**	0.418	0.249	0.093	-0.070, 0.907		0.430	0.249	0.084	-0.059, 0.919	
**Income**						-0.172	0.165	0.29774	-0.495, 0.152	

β-coefficient, SE-standard errors, CI - confidence interval, AIC - Akaike information criterion, R^2 - Adjusted R squared, Income (>$20,000)

Supplementary File: A. Details of regression supporting the association between a history of childbirth and the prevence of back pain (primary outcome variable).
B. Details of regression supporting the association between a history of childbirth and the severity of back pain (secondary outcome variable).
